# Overview of the limb injury measurement battery for quality of life (LIMB-QOL)

**DOI:** 10.1007/s11136-026-04310-z

**Published:** 2026-07-04

**Authors:** David S. Tulsky, Callie E. Tyner, Aaron J. Boulton, Pamela A. Kisala, Jerry Slotkin, Christopher L. Dearth, W. Lee Childers, Ross Zafonte, Claire Kalpakjian, Trevor Kingsbury, Seung W. Choi, Mark Sherer, Susan Bartlett, Scott Tintle

**Affiliations:** 1 Center for Health Assessment Research and Translation, University of Delaware, Newark, DE USA; 2 Departments of Physical Therapy and Psychological and Brain Sciences, University of Delaware, Newark, DE USA; 3 Extremity Trauma and Amputation Center of Excellence, Defense Health Agency, Falls Church, VA USA; 4 Department of Surgery, Uniformed Services University of the Health Sciences, Bethesda, MD USA; 5 Center for the Intrepid, Department of Rehabilitation Medicine, Brooke Army Medical Center, JBSA Ft. Sam Houston, TX USA; 6 Departments of Physical Medicine & Rehabilitation and Anesthesiology, University of Missouri School of Medicine, Columbia, MO USA; 7 Department of Physical Medicine and Rehabilitation, University of Michigan Medical School, Ann Arbor, MI USA; 8 Naval Medical Center San Diego, San Diego, CA USA; 9 University of Texas at Austin, Austin, TX USA; 10 Brain Injury Research CenterTIRR Memorial Hermann, Houston, TX USA; 11 McGill University, Montreal, Quebec, Canada; 12 Department of Orthopedic Surgery, Walter Reed National Military Medical Center, Bethesda, MD USA

**Keywords:** Health-related quality of life, Patient reported outcomes, Amputations, Traumatic, Upper extremity, Lower extremity, Limb salvage

## Abstract

**Purpose:**

The Limb Injury Measurement Battery for Quality of Life (LIMB-QOL) was developed to comprehensively assess patient reported outcomes (PRO) for research and clinical use with individuals who have sustained major extremity trauma and limb loss.

**Methods:**

A mixed-methods approach was used that included qualitative focus groups with civilians, service members, and clinicians as well as quantitative PRO data collection with individuals with a history of major traumatic limb injury or limb-loss due to such trauma or sudden-onset illness. Individuals with dysvascular and other chronic disease causes were excluded. Qualitative data were collected via focus groups (*n* = 56 individuals with major extremity trauma; *n* = 34 clinicians) and cognitive debriefing interviews (*n* = 41 individuals with major extremity trauma). Quantitative data were obtained through structured phone interviews with a large sample representing the target limb trauma and limb loss population (*n* = 603). Newly developed item banks were calibrated using graded response model item response theory analysis.

**Results:**

LIMB-QOL includes 25 measurement scales including 10 new, targeted item banks developed with and for individuals with major limb trauma and 15 existing PRO item banks measuring salient physical, emotional, and social aspects of health-related QOL. Computer adaptive tests and short forms were programmed into the Assessment Center application programming interface and are now available through REDCap and other platforms.

**Conclusion:**

LIMB-QOL provides a comprehensive and standardized system of PRO assessments for individuals with amputation or limb preservation due to sudden-onset limb injury. Research and clinical applications are discussed.

**Supplementary Information:**

The online version contains supplementary material available at https://doi.org/10.1007/s11136-026-04310-z.

## Introduction

Traumatic and sudden-onset musculoskeletal injuries are often limb-threatening, and treatable only by amputation or reconstructive surgery to preserve the limb [1, 2]. These major extremity injuries often occur instantaneously—due to traumatic causes such as motor vehicle accidents, blast injuries [3, 4], falls, or mechanical forces, or sudden-onset, non-traumatic causes such as sepsis or cancer—and have profound, long-lasting, and life-altering effects. Limb loss is associated with significant morbidity, and multiple limb loss is associated with increased mortality [5]. Limb-threatening injuries that are treated surgically to preserve or reconstruct the limb have similar functional and psychological outcomes as those treated with amputation [6–8].To better understand and document the long-term psychosocial and health-related quality of life (HRQOL) outcomes of major extremity trauma, this collaborative, multi-site research project was funded by the Congressionally Directed Medical Research Program of the U.S. Department of Defense.

The goals of this project were to: (1) identify the most important areas of function and HRQOL in people who have sustained moderate-severe limb trauma, (2) identify existing measurement instruments assessing these constructs and validate their use in this population, and (3) develop new, psychometrically sound item banks to fill any measurement gaps. This manuscript provides an overview of the methodology used in the development of the LIMB-QOL and a review of the measurement tools that comprise the battery.

### The development of the LIMB-QOL measurement system

The research team employed a systematic item banking [9] development approach as outlined in the Patient Reported Outcomes Measurement Information System (PROMIS^®^) Instrument Development and Psychometric Validation Scientific Standards [10] and as implemented in previous research by Tulsky et al. [11–15]. The LIMB-QOL development project began by engaging stakeholders to ascertain the most important factors that affect HRQOL, for individuals with major extremity trauma, and to identify topics that are inadequately covered by existing measures. New PRO item pools were developed for these areas and field tested in a large sample of individuals with limb loss and other diverse limb injuries from trauma or sudden-onset medical illness. Items were then calibrated using item response theory (IRT) [16]. In addition, for areas where existing item banks were available from PROMIS or related measurement systems, fixed forms of these measures were administered to and validated with data obtained in the LIMB-QOL sample. Each of these steps is described in more detail below.

### Domain identification and selection

#### Focus groups

HRQOL is a broad, multifaceted construct, involving physical, functional, emotional, and social aspects of life [17, 18]. Therefore, to focus and maximize relevance of any HRQOL investigation, it is critical to determine which areas of functioning are most important to the target population. For LIMB-QOL, we employed an exploratory sequential mixed-methods approach [19] for scale development. The first step focused on obtaining qualitative feedback from key stakeholders. Specifically, the study team conducted focus groups with individuals with limb loss or other major limb injuries and clinicians who work primarily with these clinical groups to identify factors affecting HRQOL that could subsequently be developed into PRO items. As described by Tyner et al. [20], the first phase of this project consisted of a series of 20 focus groups with 56 individuals with limb injuries and 34 clinicians. Trained moderators began groups with open-ended questions about the factors affecting QOL after limb injury/loss. Participants described their lives and how things had changed after injury across a variety of physical and psychosocial domains. Tyner et al. [20] found that some of the most important topics also apply to a general medical population (or could be measured by generic scales), such as pain, physical function, depression, and social participation. At the same time, several new, limb injury-specific domains were identified as important to measure. For instance, areas such as resilience and future outlook, self-esteem, body image, health-related self-efficacy, and grief regarding the loss of one’s former abilities, roles, and/or life plans were mentioned as important issues by focus group participants [20]. These data provided context for the development of new, limb injury/limb loss-targeted banks.

#### Review of existing measures

Given the goal of developing an IRT-based PRO battery, and the project team’s familiarity with large measurement systems designated as Common Data Elements [21] by the NIH (i.e., PROMIS, Neuro-QoL™, NIH Toolbox^®^, TBI-QOL™, SCI-QOL™, and SCI-FI™), the study team reviewed all IRT-based PRO banks within these existing systems to determine if the item content was relevant and appropriate for individuals with severe limb injuries/limb loss. Each item bank was reviewed in the context of the aforementioned qualitative results to determine if the overall constructs were generalizable to a limb injury population, and to assess whether some or all of the included items were appropriate for these individuals. While several existing banks were determined to be applicable to this population (e.g., PROMIS Depression and Anxiety, SCI-FI Self Care and Fine Motor), others differed from the related concept as expressed by the focus group participants (e.g., health-related self-efficacy [HRSE]) [20, 22]. In cases where existing PROMIS, Neuro-QoL, SCI-QOL, SCI-FI, and/or TBI-QOL item banks appeared to have adequate content coverage based on focus group results, fixed short forms from these item banks were moved forward to the large-scale field testing phase of the project to evaluate their validity and other psychometric properties in this population. Conversely, in areas where existing item banks were unavailable or appeared insufficient, new items were drafted—often based on focus group feedback—and refined using PROMIS qualitative item review methods (expert item review and cognitive debriefing with individuals with limb injury/limb loss). During this phase of the project, seven entirely new item banks were developed. Additionally, three existing item banks (SCI-QOL Resilience, Grief/Loss, and Self-Esteem) were determined to be appropriate but lacked sufficient content coverage for individuals with limb loss/limb trauma and were therefore supplemented by newly written items. These revised measures were subsequently treated as new item pools, field tested, and calibrated using IRT. Ultimately, 10 new item pools and items from 6 PROMIS measures, 5 Neuro-QoL measures, 7 SCI-QOL/SCI-FI measures, and an additional disability-specific measure of financial hardship designed for use in rehabilitation populations (see Supplemental Table 1) were planned for field testing as part of the LIMB-QOL measurement battery.

#### New item writing

**Fig. 1:**
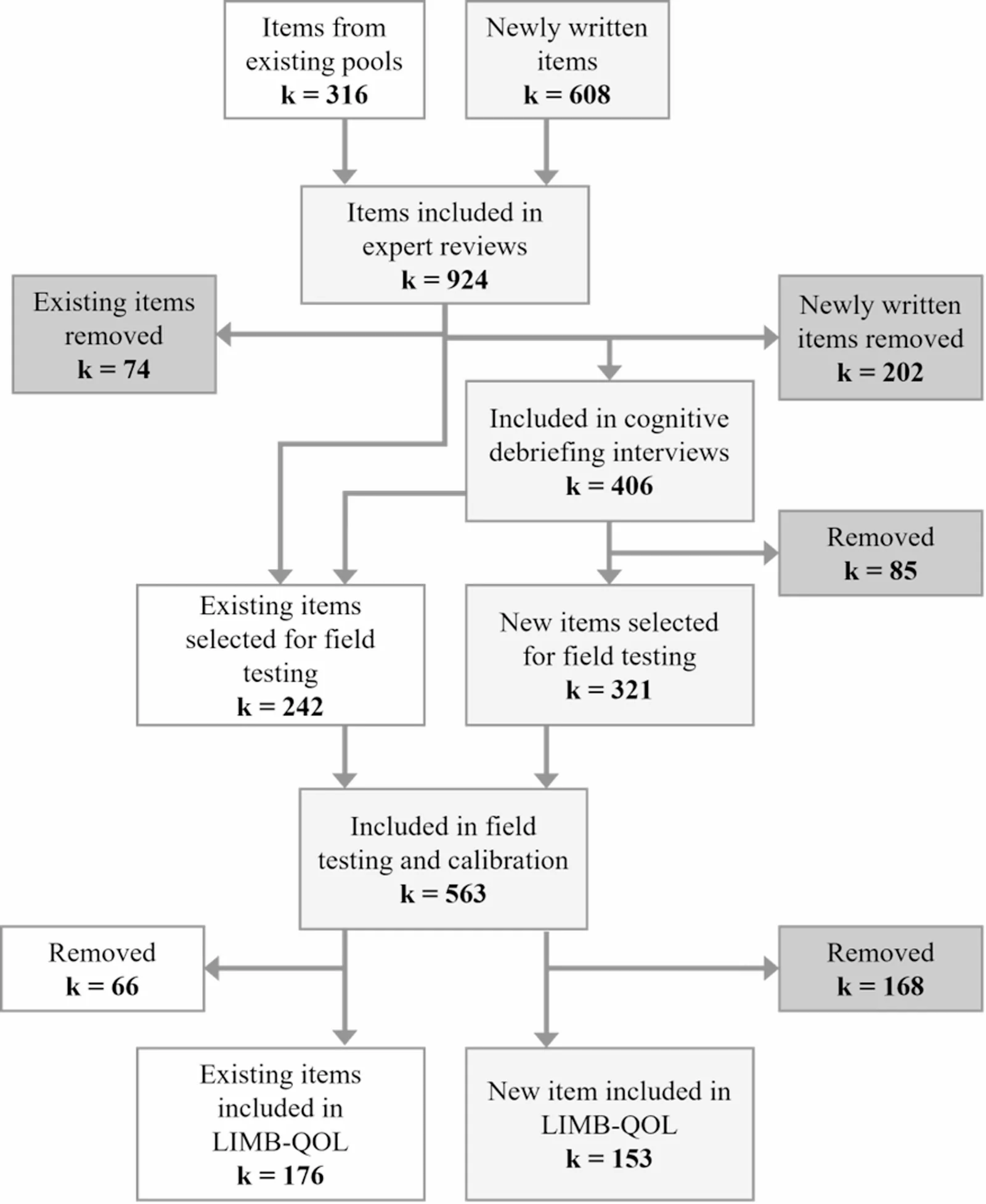
Flow chart of item development process. *Notes*: Items from existing pools includes items from PROMIS, Neuro-QoL, SCI-QOL, SCI-FI, and/or TBI-QOL item banks. Items included in cognitive debriefing interviews were all new, except for 7 items from the TBI-QOL military-related loss and guilt item banks developed by Toyinbo et al. [23]. SCI-QOL, spinal cord injury quality of life measurement system. TBI-QOL, traumatic brain injury quality of life measurement system. SCI-FI/C, spinal cord injury functional index/capacity. Neuro-QoL, quality of life in neurological disorders. PROMIS, patient-reported outcomes measurement information system

For the HRQOL domain areas that needed new item development (e.g., to fill the identified HRQOL measurement gaps), new items were drafted based on focus group feedback and input from expert clinicians and researchers. In some cases, verbatim focus group quotes formed the basis of new item content (e.g., “Early on, before I had a prosthetic leg…I was embarrassed to go out and about” became “I felt embarrassed about my appearance”). Eleven new item pools were compiled for a total of 608 preliminary items. These items and associated construct definitions then underwent multiple rounds of review by the study team and external experts in preparation for review from people with limb loss or major limb injuries. Within each subdomain, items were arranged by difficulty, and each pool was assessed for construct representativeness. Reviewers were instructed to flag items that were redundant, irrelevant, unclear, or otherwise problematic; and to identify any topics that were missing. Through this process, the item pools were narrowed to 406 new items to be brought forward for the next phase of development (see Fig. 1).

#### Qualitative item review: cognitive debriefing interviews

Cognitive debriefing interviews—a qualitative approach that gathers input on the question answering process from the perspective of stakeholders [24, 25]—were conducted on all newly written items. Individuals with limb loss/injury responded to each item and were then asked to describe their rationale and thought process in selecting an answer. Through a series of standardized verbal probes, participants were asked to comment on the relevance, importance, comprehensibility, and clarity of the items, and on their personal decision-making and response processes. Cognitive debriefing helps ensure that each item and its associated response options are probing for the content that the test developers intended, without unintended associations or confounds. Overall, cognitive debriefing serves to enhance the content validity of the items.

Forty-one individuals with limb injury (see Table 1 for detail on cognitive interview participant characteristics) recruited from three military treatment facilities (MTFs) and two civilian medical centers reviewed drafted items. Given the large number of draft items, the items were divided into smaller subsets of 40–80 items, with at least five participants reviewing each subset of items. All interviews were conducted by telephone by Masters-level research assistants (RAs). RAs were trained and certified in cognitive debriefing techniques by a study investigator (CT). Participants’ responses and any spontaneous feedback were recorded by the RA in a dedicated REDCap database. Following synthesis and review of interview feedback, the wording of most items was deemed appropriate. Eighty-five items were removed (primarily due to redundancy with other items) and 33 items were reworded based on participant feedback (e.g., “*I felt comfortable with my appearance*” was changed to “I felt at ease with my appearance” due to the possible physical connotation of comfort/discomfort). Following these modifications, the final 321 new items across 11 domains (see Supplemental Table 1) were administered to a large sample of individuals with limb injury to facilitate development of new, targeted item banks as a component of LIMB-QOL.

**Table 1:** Participant demographics and injury characteristics

	Cognitive debriefing		Large-scale field-testing							
			Calibration study (T1)		Validation study (T2)		Test-retest study (T3)		Long-term follow-up (T4)	
	*N* = 42		*N* = 603		*N* = 204		*N* = 160		*N* = 356	
	M	(SD)	M	(SD)	M	(SD)	M	(SD)	M	(SD)
Age (years)	38.3	(11.6)	41.8	(13.1)	41.9	(13.2)	41.6	(12.8)	41.9	(13.2)
Time since injury (years)	9.8	(10.9)	8.7	(8.9)	9.9	(10.2)	10.5	(10.6)	8.8	(9.2)

	n	(%)	n	(%)	n	(%)	n	(%)	n	(%)
Gender										
Male	35	(83.3)	471	(78.1)	155	(76.0)	128	(80.0)	282	(79.2)
Female	7	(16.7)	130	(21.6)	48	(23.5)	31	(19.4)	72	(20.2)
Other	0	(0.0)	1	(0.2)	0	(0.0)	0	(0.0)	1	(0.3)
Do not wish to provide	0	(0.0)	1	(0.2)	1	(0.5)	1	(0.6)	1	(0.3)
Ethnicity										
Non-hispanic/latino	38	(90.5)	522	(86.6)	172	(84.3)	137	(85.6)	300	(84.3)
Hispanic/latino	4	(9.5)	79	(13.1)	31	(15.2)	23	(14.4)	54	(15.2)
Do not wish to provide	0	(0.0)	2	(0.3)	1	(0.5)	0	(0.0)	2	(0.6)
Race										
White	34	(81.0)	471	(78.1)	160	(78.4)	124	(77.5)	278	(78.1)
Black or African American	3	(7.1)	55	(9.1)	16	(7.8)	11	(6.9)	37	(10.4)
Asian	0	(0.0)	13	(2.2)	4	(2.0)	4	(2.5)	5	(1.4)
Native Hawaiian or Other Pacific Islander	0	(0.0)	5	(0.8)	3	(1.5)	3	(1.9)	5	(1.4)
American Indian/Alaskan Native	0	(0.0)	4	(0.7)	1	(0.5)	1	(0.6)	2	(0.6)
Other race	4	(9.5)	32	(5.3)	9	(4.4)	8	(5.0)	17	(4.8)
Multiple races	1	(2.4)	20	(3.3)	10	(4.9)	8	(5.0)	9	(2.5)
Do not wish to provide	0	(0.0)	3	(0.5)	1	(0.5)	1	(0.6)	3	(0.8)
Injury location										
Upper limb	6	(14.3)	114	(18.9)	44	(21.6)	28	(17.5)	64	(14.6)
Lower limb	32	(76.2)	412	(68.3)	128	(62.7)	109	(68.1)	240	(67.4)
Both upper and lower limbs	4	(9.5)	77	(12.8)	32	(15.7)	23	(14.4)	52	(14.6)
Limb treatment status ^a^										
Limb loss/amputation	40	(95.2)	419	(69.5)	148	(72.5)	122	(76.2)	255	(71.6)
Surgical preservation/reconstruction only	2	(4.8)	184	(30.5)	56	(27.5)	38	(23.8)	101	(28.4)
Trauma status ^b^										
Traumatic	42	(100.0)	482	(79.9)	171	(83.8)	138	(86.2)	295	(82.9)
Non-traumatic only	0	(0.0)	121	(20.1)	33	(16.2)	22	(13.8)	61	(17.1)
Military status										
Active-duty ^c^	10	(23.8)	129	(21.4)	47	(23.0)	39	(24.4)	92	(25.8)
Veteran	12	(28.6)	186	(30.8)	67	(32.8)	60	(37.5)	108	(0.3)
Civilian	20	(47.6)	288	(47.8)	90	(44.1)	61	(38.1)	156	(43.8)

^a^ Limb status defined as the loss of at least one major extremity due to a qualifying injury; ^b^ Trauma status defined as at least one qualifying major extremity injury having occurred due to a traumatic cause; ^c^ Includes military reservists. Due to rounding, percentages within categories may not add to 100

### Large-scale field testing

Participants for the large-scale field testing were recruited from three MTFs and six civilian medical centers, with a goal of obtaining a heterogeneous sample with regard to treatment (amputation versus limb reconstruction/preservation) injury location (upper versus lower limb involvement), and history of military service (Service members/Veterans versus civilians). Individuals were eligible to participate if they met all three of the following criteria: (1) ability to speak, read, and understand English; (2) age 18 to 65 years at the time of study enrollment, and (3) have a history of at least one *traumatic injury* or *sudden-onset acquired disability* affecting a major extremity. Injury was defined as physical damage from sudden or brief exposure to intolerable levels of energy (e.g., mechanical, electrical, thermal, or chemical forces). Examples of sudden-onset acquired disability include bone cancer, complications of medical or surgical care, compartment syndrome, or infections leading to loss of a limb or permanent functional impairment/disability of one or more major extremities (i.e., involving the arm and being at or proximal to the wrist, and/or involving the leg and being at or proximal to the ankle joint; injuries resulting in partial hand or foot loss were included, although injuries affecting only finger(s)/toe(s) were excluded). Finally, at least one affected major extremity must have (a) been amputated or (b) resulted in a persistent functional deficit and/or been treated surgically for the purpose of preserving/reconstructing the injured limb. Individuals with major extremity trauma due to a high velocity injury (e.g., gunshot wound, blast injury) were automatically included. Participants were excluded if (1) they presented with cognitive or mental health problems that would interfere with completion of the study, (2) their qualifying injury was related to a progressive or degenerative disease (e.g., due to dysvascular causes), or (3) for individuals *without* limb loss, *only one* of the following was affected: tendon, ligament, nerve, muscle, bone, or artery (i.e., involvement of two or more of these systems was required to participate). Eligibility was confirmed through medical record review, and the institutional review board at each site reviewed and approved the research.

All data collection was conducted by phone interview. Each interview consisted of demographic and health history questions and up to 636 PRO items, as relevant to the participant (for example, only participants who use an orthosis/prosthesis were given questions about orthosis/prosthesis use). Additional questions related to COVID-19 impact were added (with IRB approval) during the pandemic.

Interviews typically lasted about 2 h; participants were given the option to complete the interview across two sessions as long as both sessions were completed within 7 calendar days. On rare occasions, the interview was divided across more than two sessions (*n* = 14; 2.3% of the sample). Since all item calibration analyses were to be conducted within, rather than across, item pools, when interviews were paused, the pause was *between* item pools, such that individual participants completed all the items in a pool at a single point in time to minimize any chance of change in the underlying trait (and therefore potential error in parameter estimates). While the goal was to complete all questions within a 7 day span (*n* = 514 or 85.2% of the sample completed the study within a week), 89 participants (14.8% of the sample) needed additional time to reschedule, with the vast majority (*n* = 67) completing the questions within 14 days.

To ensure standardization of methods and administration, all data collectors were Master’s-level RAs who were trained and certified on administration procedures. Prior to their first interview, each RA completed multiple independent practice sessions and a final certification session with a project Co-Investigator (CT). Further, as part of the standardized interview procedures and to ensure that participants were considering the appropriate response set for each item, the study employed color-coded response cards that were provided to participants (by postal mail or email) prior to the interview session. The interviewer script was embedded in the REDCap interview and contained prompts to direct the participant to the appropriate response card for each section of the interview. In this way, the participant was always looking at the appropriate response set as each item was read aloud. All participant responses were entered directly into the study REDCap database by the interviewer. Weekly supervision meetings were held with all RAs performing data collection by project Co-Investigators (CT and JS) to ensure fidelity to study procedures and to troubleshoot any issues.

#### Test-retest, validation, and follow-up

A subset of the participants from the large-scale field testing were recruited to participate in several follow-up assessments. These included a pair of interviews that occurred after one year (T2 for 1-year follow-up and T3 for 1-week retest) to assess the construct validity and test-retest reliability of the new measures (see Supplemental Table 2). A fourth interview occurred two to three years following baseline (T4) to capture long-term follow-up. Each interview was conducted using the same procedures and RAs as described for the baseline interviews. At the T2 interview, participants completed all measures from baseline as well as a set of criterion measures—at least one per each new LIMB-QOL item pool. Characteristics of participants who completed the T2 interview (*n* = 204) are shown in Table 1 (Validation Study). Two strategies were employed to accommodate the additional burden caused by adding the criterion measures to the interview protocol. First, short forms of each new LIMB-QOL item pool were administered. Participants then completed a subset of the criterion measures that were selected via a randomized testing design; specifically, participants were randomized to complete one of two possible blocks of the criterion measures (a limited number of criterion measures with pre-existing filter logic, such as use of an orthotic/prosthetic device, were completed by all qualifying participants; see Supplemental Table 2). A subset of the follow-up participants completed the T3 interview (*n* = 160; Test-Retest Study in Table 1), which contained only the LIMB-QOL short forms and occurred 7–14 days after the T2 interview.

The sampling goals for the T2 interviews were dictated by the number needed to achieve sufficient power for validity analyses, and for the T3 interviews the sample size was focused on the power needed for test-retest reliability analyses. For the T4 interviews, which occurred two to three years after baseline, the sampling goal was to include as many participants as possible from the original study to be able to track change over time and to maintain contact with this large, well-characterized cohort. To this end, 59% of the original sample (*n* = 356) completed a long-term follow-up (T4) interview, which we anticipate will allow for future research to define the patterns of HRQOL seen in this clinical population over time.

### Data analysis

**Fig. 2:**
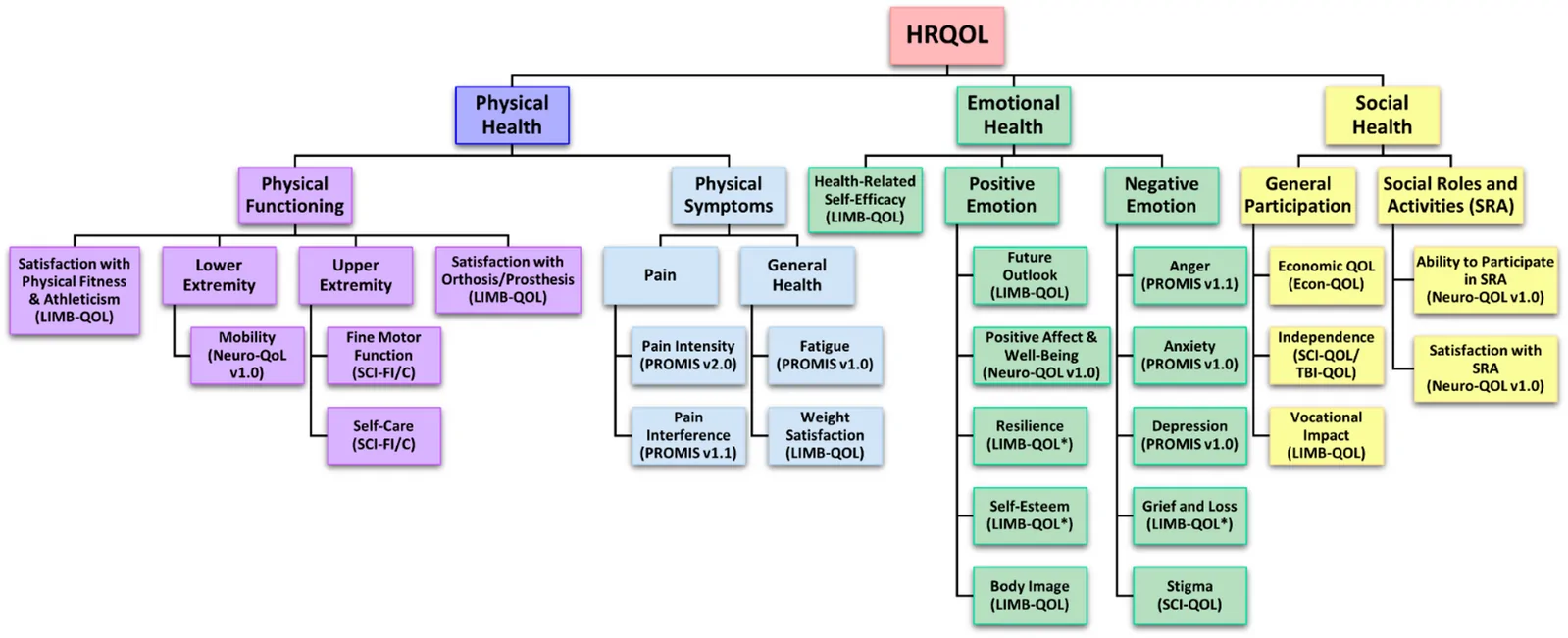
Framework of HRQOL category coverage for the LIMB-QOL measures. *Notes*: HRQOL, health-related quality of life. LIMB-QOL, limb injury measurement battery for quality of life newly developed measures. LIMB-QOL*, measures originally developed as part of SCI-QOL and/or TBI-QOL that were recalibrated for LIMB-QOL. SCI-QOL, spinal cord injury quality of life measurement system. TBI-QOL, traumatic brain injury quality of life measurement system. SCI-FI/C, spinal cord injury functional index/capacity measures, which are part of the SCI-QOL measurement system. Neuro-QoL, quality of life in neurological disorders measures included for use with LIMB-QOL. PROMIS, patient-reported outcomes measurement information system measures included for use with LIMB-QOL

The psychometric and statistical aspects of data analysis and IRT calibration are described in detail in Boulton et al. [26]. Briefly, results supported the creation of a targeted battery of measures for individuals with limb loss/injury (see Fig. 2). Eight new IRT-calibrated item banks (5 of which contain entirely new content and 3 of which were adapted from SCI-QOL or TBI-QOL and recalibrated in the LIMB-QOL field testing sample) and 2 new fixed-length scales were completed (see Table 2). Fifteen existing PROMIS, Neuro-QoL, SCI-QOL, TBI-QOL, or SCI-FI item banks (see Table 3) were also selected for inclusion in the LIMB-QOL. For the most part, data on existing measures provided strong support for their validation in a limb injury sample and therefore for inclusion in LIMB-QOL. Key reliability results for these 10 new measures – internal consistency, test-retest reliability, and conditional reliability – are provided in Table 4.

**Table 2:** Overview of new LIMB-QOL measures

Category	Domain	# Items	CAT available?	# Short form items	Scoring direction	Item context
Physical functioning	Satisfaction with physical fitness and athleticism	15	Yes	7	Better function	In the past 7 days
	Satisfaction with orthosis/prosthesis	15	Yes	7	Better function	In the past 7 days
Emotional health	Body image	29	Yes	9	Better function	In the past 7 days
	Future outlook	21	Yes	8	Better function	N/A
	Grief and loss	25	Yes	8	Worse symptoms	In the past 7 days
	Health-related self-efficacy	16	Yes	7	Better function	Lately
	Resilience	40	Yes	9	Better function	Lately
	Self-esteem	34	Yes	9	Better function	In the past 7 days
	Weight satisfaction	10	No	–	Better function	Lately
Social participation	Vocational impact	7	No	–	Worse function	Lately

All measures use the response set of never/rarely/sometimes/often/always

**Table 3:** Domain definitions and reference population for LIMB-QOL measures by category

Category	Domain	Definition	Reference population
Physical functioning	Satisfaction with physical fitness and athleticism	Individuals’ satisfaction and feelings about their physical abilities, including physical fitness and sporting activities. Higher scores indicate greater satisfaction with physical fitness and athleticism. Note: This domain does not measure specific activities (e.g., ability to walk a specified distance, running).	LIMB-QOL
	Satisfaction with orthosis/prosthesis	Individuals’ satisfaction with, and emotional and physical assimilation of, an orthosis and/or prosthesis. This includes satisfaction with device performance and aesthetics as well as user adaptation to a device, including usage, fit, and comfort. Higher scores indicate greater satisfaction with one’s orthosis/prosthesis. Note: This pool is specific to individuals who use orthotic and/or prosthetic devices.	LIMB-QOL
	Lower extremity function/mobility (Neuro-QoL v1.0)	One’s ability to carry out various activities involving the trunk region and increasing degrees of bodily movement, ambulation, balance, or endurance [27].	Clinical (linked to Neuro-QoL metric)
	Self-care (SCI-FI/C)	Measures an individual’s ability to perform daily self-care activities such as eating, dressing, grooming, and bathing [13, 28].	Clinical (SCI)
	Fine motor function (SCI-FI/C)	Assesses various components of fine motor functioning including the ability to manually hold, manipulate and move objects that require varying degrees of dexterity and/or strength [13, 28].	Clinical (SCI)
Physical symptoms	Fatigue (PROMIS v1.0)	Measures a range of symptoms, from mild subjective feelings of tiredness to an overwhelming, debilitating, and sustained sense of exhaustion that likely decreases one’s ability to execute daily activities and function normally in family or social roles [29].	General population
	Weight satisfaction	The Weight Satisfaction Scale measures an individual’s feelings and self-appraisal about their current body weight. Higher scores indicate more satisfaction with one’s weight.	LIMB-QOL
	Pain interference (PROMIS v1.1)	Measures the consequences of pain including the extent to which pain hinders engagement with social, cognitive, emotional, physical and recreational activities [30].	General population
	Pain intensity (PROMIS v2.0)	Measures how much a person hurts [29].	General population
Emotional health	Body image	Self-appraisal of physical appearance including feeling self-conscious or otherwise uncomfortable with one’s body appearance and concerns about how others perceive one’s body appearance. Some items in this pool are specific to the way an individual’s amputation, residual limb, and/or preserved limb affects this appraisal. Higher scores indicate a more positive body image. Note: This item pool does not contain items pertaining to orthosis/prosthesis. Additionally, items pertaining to body weight are covered under the Weight Satisfaction scale.	LIMB-QOL
	Future outlook	One’s future orientation including planning, goal-setting, and levels of optimism/pessimism. Higher scores indicate a more positive outlook for the future.	LIMB-QOL
	Health-related self-efficacy	A person’s self-reported engagement and agency in managing their health and participating in their ongoing recovery. Higher scores indicate greater perceived health-related self-efficacy.	LIMB-QOL
	Grief and loss^a^	Reactions to loss associated with the limb injury. The items assess experience of emotional reactions to injury-related losses, such as sadness, regret, and loss of identity. Higher scores indicate more problems with grief and loss.	LIMB-QOL
	Resilience^a^	A person’s degree of acceptance, adaptation, and emotional endurance following limb injury. Included items address motivation, positive reappraisals, and effective coping. Higher scores indicate a greater degree of resilience.	LIMB-QOL
	Self-esteem^a^	Self-confidence and self-acceptance. Items in this domain focus on self-appraisal (both positive and negative), as well as beliefs about how others perceive oneself. Higher scores indicate higher self-esteem. Note: Self-esteem related solely to physical appearance is categorized as Body Image.	LIMB-QOL
	Anger (PROMIS v1.1)	Self-reported angry mood (irritability, frustration), negative social cognitions (interpersonal sensitivity, envy, disagreeableness), and efforts to control anger [29].	General population
	Anxiety (PROMIS v1.0)	Fear (fearfulness, panic), anxious misery (worry, dread), hyperarousal (tension, nervousness, restlessness), and somatic symptoms related to arousal (racing heart, dizziness) [29].	General population
	Depression (PROMIS v1.0)	Negative mood (sadness, guilt), views of self (self-criticism, worthlessness), and social cognition (loneliness, interpersonal alienation), as well as decreased positive affect and engagement (loss of interest, meaning, and purpose) [29].	General population
	Positive affect and well-being (Neuro-QoL v1.0)	Aspects of a person’s life that relate to a sense of well-being, life satisfaction, or an overall sense of purpose and meaning [27].	General population
	Stigma (SCI-QOL)	Refers to negative stereotyping that leads to discrimination. […] The SCI-QOL Stigma item bank assesses perceptions of self and publicly enacted negativity, prejudice, and discrimination as a result of injury manifestations [14].	Clinical (linked to Neuro-QoL metric)
Social participation	Vocational impact	Effect someone’s limb injury has had on their work experiences and/or career. Higher scores indicate higher negative impact of the injury.	LIMB-QOL
	Ability to participate in SRA (Neuro-QoL v1.0)	Degree of involvement in one’s usual social roles, activities, and responsibilities; including work, family, friends, and leisure [27].	General Population
	Satisfaction with SRA (Neuro-QoL v1.0)	Satisfaction with involvement in one’s usual social roles, activities, and responsibilities; including work, family, friends, and leisure [27].	General Population
	Independence (SCI-QOL/TBI-QOL)	Refers to perceived independence or ability to communicate one’s needs and sense of control over one’s life [14].	Clinical (SCI)
	Economic QOL	This measure of economic and financial QOL relevant to individuals with disabilities includes items assessing one’s ability to afford needed items or services, opportunities for income, and anxiety about money [31].	Clinical (SCI, TBI, Stroke)

^a^Modified from SCI-QOL/TBI-QOL and recalibrated in a limb injury sample

**Table 4:** Internal consistency, test-retest reliability, and conditional reliability for new LIMB-QOL measures

Domain			T scores								
	α	Test-retest ICC	10	20	30	40	50	60	70	80	90
*Physical functioning*											
Satisfaction with physical fitness and athleticism	0.95	0.86	0.34	0.75	0.92	0.95	0.96	0.95	0.90	0.66	0.22
Satisfaction with orthosis/prosthesis	0.96	0.77	0.54	0.85	0.96	0.97	0.96	0.90	0.62	0.21	0.04
*Emotional health*											
Body image	0.98	0.84	0.72	0.95	0.98	0.98	0.98	0.95	0.75	0.34	0.08
Future outlook	0.97	0.86	0.84	0.97	0.97	0.97	0.97	0.96	0.77	0.32	0.08
Grief and loss	0.98	0.83	0.01	0.09	0.51	0.92	0.98	0.99	0.98	0.92	0.49
Health-related self-efficacy	0.94	0.68	0.88	0.95	0.95	0.95	0.94	0.84	0.52	0.19	0.05
Resilience	0.98	0.81	0.95	0.98	0.98	0.98	0.98	0.97	0.90	0.64	0.26
Self-esteem	0.98	0.87	0.86	0.98	0.99	0.99	0.98	0.96	0.77	0.31	0.06
Weight satisfaction	0.95	0.91	0.39	0.82	0.94	0.96	0.96	0.94	0.67	0.16	0.02
*Social participation*											
Vocational impact	0.94	0.80	0.01	0.08	0.51	0.91	0.95	0.95	0.80	0.23	0.02

Internal consistency (α) computed at T1. Test-retest reliability (ICC) computed between T2 and T3. Conditional reliability estimates computed using each measure’s test information function. The values also demonstrate the range of scores for which each measure is the most precise

## Domain descriptions

As seen in Table 3, LIMB-QOL is comprised of 25 subdomains across four dimensions of physical functioning, physical symptoms, emotional health, and social participation. Information on item context (timeframe) and Likert-type response options for the new LIMB-QOL measures are presented in Table 2. Subdomain descriptions for all newly developed and adapted/re-calibrated LIMB-QOL item banks, fixed-length scales, and LIMB-QOL measures from existing systems are included as Table 3.

### Scoring and administration

The eight new IRT-calibrated item banks are available either as CATs or as brief, fixed-length short forms. For measures that are part of the PROMIS, Neuro-QoL, or SCI-QOL/SCI-FI, only short form versions were field tested.

All measures in LIMB-QOL are scored on a T-score metric with a mean of 50 and a standard deviation of 10, in reference to the LIMB-QOL calibration field-testing sample (*n* = 603; see Table 1). As seen in Table 3, the 15 existing measures reference either the general population or clinical samples; for the 4 measures based on a clinical SCI, TBI, or stroke reference sample (Self-Care, Fine Motor, Independence, Econ-QOL), users may wish to use raw (sum) scores instead of T scores. For all measures, the scoring direction is such that higher scores indicate more of the trait being measured. Therefore, for “positively” named measures such as Body Image or Resilience, higher scores indicate better function, whereas for “negatively” named measures such as Grief/Loss, higher scores indicate worse symptoms (see Table 2).

All new and existing LIMB-QOL measures are available through the Assessment Center [32] application programming interface, which allows their access through the public REDCap [33, 34] instrument library. To locate the measures in the REDCap Shared Library, the keywords “limb” or “LIMB-QOL” may be used. In REDCap nomenclature, CATs are marked as “Adaptive Instruments,” and scored short forms are marked as “Auto-scoring.” Users wishing to access a stand-alone digital copy of the item banks or short forms may contact the authors at LIMB-QOL@udel.edu.

## Discussion

This LIMB-QOL development project has established a comprehensive system of HRQOL measures covering multiple dimensions of functioning for use with individuals with moderate to severe limb injuries and limb loss. While other work has validated individual measures (or subsets of measures) for individuals with orthopedic injuries [35–37], and measurement batteries have been developed specifically for lower-extremity trauma [38–41], LIMB-QOL is the first to formally specify and validate a standardized set of existing PROs complemented by the development of new, limb-injury-specific IRT-calibrated item banks and scales applicable to individuals with either upper or lower major extremity injury. Notably, stakeholders with limb trauma and limb loss were included at every step of measure development and validation. Furthermore, inclusion of feedback from expert clinicians and researchers throughout the project has ensured that clinically relevant issues were not overlooked. Another strength of this work is that LIMB-QOL involved a large and diverse sample of participants with upper and/or lower extremity injuries, both traumatic and non-traumatic, military and civilian, as well as those whose injuries were treated by amputation or limb preservation/reconstruction. This sample reflects the real-world population often seen in rehabilitation settings, and ensures that the measurement battery is relevant and appropriate for a wide variety of individuals who have had catastrophic injuries or sudden-onset medical conditions affecting the integrity of their limbs. The extensive coverage of multiple dimensions of functioning is an important innovation of this project, given that most research in HRQOL outcomes in orthopedic populations focuses within more limited clinical categories.

One goal in developing the LIMB-QOL as a battery, or set of measures pulled from multiple sources, was to create a user-friendly set of measures that would be easily accessible and could be utilized by researchers and clinicians in orthopedic settings who are already using PROMIS and related measures such as Neuro-QoL, SCI-QOL, or TBI-QOL. Such users would already be familiar with the administration and scoring procedures used for LIMB-QOL [37, 42]. The new, limb-injury-specific measures allowed us to fill critical measurement gaps while retaining PROMIS-type characteristics, such as the T-score metric and item contexts and response sets. As detailed in Boulton et al. [26], our methodology included rigorous item bank calibration techniques, including systematic examination of items both individually and collectively (e.g., by replicating PROMIS techniques for identifying and eliminating multidimensionality) and a graded-response IRT model. Furthermore, co-administering the existing banks to our calibration field testing sample alongside the new measures has allowed us to provide evidence of the validity of these measures for individuals who have sustained severe limb injuries. Finally, a significant benefit of the battery approach to PRO measurement is that many of the included PROMIS and Neuro-QoL measures have already been translated into a multitude of other languages [43, 44], thus greatly reducing the amount of future work needed to translate the overall LIMB-QOL battery.

## Study limitations and future directions

All LIMB-QOL field testing items were administered in a structured interview format by trained and certified data collectors, in contrast to independent self-report via computer, tablet, or paper-and-pencil form. While this may be viewed as a limitation, our previous research has demonstrated that the mode of data collection—specifically examining structured interview versus computer self-report—has a minimal-to-negligible effect on participant responses [45] and should not impact the findings of this study. In fact, we have previously found that data collected in a highly structured interview format improves accessibility for participants who may have difficulty navigating a computer independently due to upper extremity functional limitations. Additionally, it can increase participant engagement, allowing for significantly longer interview sessions and less missing data [11]. Furthermore, In this study, all data were collected by interviewers trained to administer the questionnaires and followed the methodology honed in previous research, such as the SCI-QOL and TBI-QOL development studies [46].

Further, administration of the full LIMB-QOL battery currently produces 25 distinct subdomain scores. Future factor analytic work aggregating variables into composite scores will reduce the complexity of scoring and interpreting the LIMB-QOL battery. Future research can also examine different patterns of scores among subgroups of individuals, for example for those with upper- versus lower-extremity injuries.

Finally, we anticipate that systematic use of LIMB-QOL in clinical and research settings will improve the field’s understanding of HRQOL after limb injury, although future research is necessary. Repeated longitudinal assessments/monitoring could improve understanding of HRQOL outcomes over time and could inform future intervention for individuals with limb injuries. We believe that future analyses of symptoms and functioning across domains could help identify symptom clusters [47], which can potentially help inform primary and secondary targets for intervention.

## Conclusion

LIMB-QOL represents a conceptually relevant, psychometrically sound, and brief yet comprehensive way to assess the most relevant domains affecting HRQOL in individuals with major extremity trauma or sudden-onset acquired disability. We anticipate that LIMB-QOL will be particularly valuable for longitudinal assessment and for the evaluation of intervention outcomes. LIMB-QOL is readily available to any researchers or clinicians wishing to assess HRQOL outcomes using PROs through the REDCap Shared Library or can be obtained by contacting LIMB-QOL@udel.edu.

## Supplementary Information

Below is the link to the electronic supplementary material.

Supplementary Material 1.

## Data Availability

The data presented in this article are not readily available because a data use agreement must be signed prior to release. Requests to access the datasets should be directed to LIMB-QOL@udel.edu.
