# Supplementary material for: Overview of the limb injury measurement battery for quality of life (LIMB-QOL)
Source: Qual Life Res. 2026 Jul 4;35(8):224. doi: 10.1007/s11136-026-04310-z (PMC13332998; doi:10.1007/s11136-026-04310-z)
Supplement: Supplementary file 1 — Supplementary Material 1. [file 11136_2026_4310_MOESM1_ESM.docx]

**Supplementary Information**

**Overview of the Limb Injury Measurement Battery for Quality of Life (LIMB-QOL)**

***Journal****: Quality of Life Research*

Tulsky, D.S., Tyner, C.E., Boulton, A.J., Kisala, P.A., Slotkin, J., Dearth, C.L., Childers, W.L., Zafonte, R., Kalpakjian, C., Kingsbury, T., Choi, S. W., Sherer, M., Bartlett, S., & Tintle, S.

Corresponding Author: David Tulsky, Ph.D., University of Delaware, [dtulsky@udel.edu](mailto:dtulsky@udel.edu)

***Supplemental Table 1. Measures Administered During LIMB-QOL Field Testing***

| **Newly Developed Item Pools** | **Administered at Baseline** | **Administered at T2, T3, T4** |
| --- | --- | --- |
| Body Image | 31-item pool | SF 8b |
| Resilience | 60-item pool | SF 9a |
| Future Outlook | 23-item pool | SF 8a |
| Grief & Loss | 32-item pool | SF 7b |
| Self-Esteem | 42-item pool | SF 9a |
| Satisfaction with Orthosis/Prosthesis | 65-item pool | SF 4b |
| Vocational Impact | 24-item pool | SF 6b |
| Satisfaction with Physical Fitness & Athleticism | 36-item pool | SF 6b |
| Health-Related Self-Efficacy | 38-item pool | SF 4b |
| Weight Satisfaction | 26-item pool | 10-item scale |
| Adjustment to Military Identity/Role Change ^a^ | 51-item pool | 16 items from pool |
| **Other Measures that are Part of LIMB-QOL** | **Administered at Baseline** | **Administered at T2, T3, T4** |
| Pain Interference (PROMIS v1.1) | SF8a | SF8a |
| Pain Intensity (PROMIS v2.0) | SF3a | SF3a |
| Fatigue (PROMIS v1.0) | SF7a | SF7a |
| Independence (SCI-QOL v1.0 and TBI-QOL v.1.0) | SF8a | SF8a |
| Lower Extremity Function/Mobility (Neuro-QoL v1.0) | SF8a | SF8a |
| Fine Motor (SCI-FI/C) | SF9a | SF9a |
| Self-Care (SCI-FI/C) | SF11a | SF11a |
| Depression (PROMIS v1.0) | SF8b | SF8b |
| Anxiety (PROMIS v1.0) | SF7a | SF7a |
| Anger (PROMIS Item Bank v1.1) ^b^ | Custom SF | Custom SF |
| Positive Affect and Well-Being (Neuro-QoL v1.0) | SF9a | SF9a |
| Ability to Participate in SRA (Neuro-QoL v1.0) | SF8a | SF8a |
| Satisfaction with SRA (Neuro-QoL v1.0) | SF8a | SF8a |
| Stigma (Neuro-QoL v1.0) | SF8a | SF8a |
| Economic Quality of Life v2.0 ^c^ | SF8a | SF8a |
| **Criterion Measures** | **Administered at Baseline** | **Administered at T2, T3, T4** |
| Upper Extremity Function (Neuro-QoL v1.0) ^d^ | SF8a | SF8a |
| Perceived Stress (NIH Toolbox v2.0) | 10-item FF | 10-item FF |
| Social Isolation (PROMIS v2.0) | SF8a | SF8a |
| Loneliness (NIH Toolbox v2.0) | 5-item FF | 5-item FF |
| Executive Function (Neuro-QoL v1.0) | SF8a | SF8a |
| Cognition – General Concerns (Neuro-QoL v1.0) | SF8a | SF8a |
| Health-Related Self-Efficacy for Managing Chronic Conditions – Managing Medications and Treatment (PROMIS Item Bank v1.0) ^b^ | Custom 11-item SF | Custom 11-item SF |
| PTSD Checklist (PCL/PTSD-5) | 6-item FF | 6-item FF |

Notes: SF=Short Form. FF=Fixed Form. SRA = Social Roles and Activities. Short-form “a” versions refer to the primary short forms developed following baseline testing and IRT calibration. Short-form “b” versions refer to secondary versions created before final calibrations were available, and due to participant burden, shortened versions of each item pool were administered). ^a^ This item pool has not been calibrated due to the limited sample size of individuals who were Service members or Veterans. ^b^ Only a subset of items from this form/item bank was included in field testing. ^c^ Economic Quality of Life is a standalone measure calibrated with individuals with SCI, TBI, and stroke, and published by Tulsky *et al.* (2015) [31]. The 8-item version 2.0 [48] was used for data collection. ^d^ Upper Extremity Function was considered but ultimately not included in LIMB-QOL.

***Supplemental Table 2. Additional Validation Measures Administered at Field Testing T2***

| **Measure Name** | **Block** | **Notes on Administration** |
| --- | --- | --- |
| Body Esteem Scale for Adolescents and Adults (BESAA) [49] | Arm 1 | Included the Body Image Weight Scale (8 items) and the Appearance Scale (10 items) |
| Rosenberg Self-Esteem Scale (RSES) [50] | Arm 1 |  |
| Modified Weight Bias Internalization Scale (WBIS-M) [51] | Arm 1 |  |
| Internal Fitness Scale (IFS) [52] | Arm 1 |  |
| Multidimensional Health Locus of Control Scales (MHLC), Form B [53]^h^ | Arm 1 | Consisted of Internal Beliefs, Chance Beliefs, and Powerful Others subscales (6 items each) |
| Connor-Davidson Resilience Scale 25 (CD-RISC) [54] | Arm 2 |  |
| State Optimism Measure (SOM) [55] | Arm 2 |  |
| Life Orientation Test – Revised (LOT-R) [56] | Arm 2 |  |
| The Loss Inventory [57] | Arm 2 |  |
| Knee Injury and Osteoarthritis Outcome Score (KOOS) [58] | Both | Skipped if participant is unable to walk. |
| PROMIS Global Health Scale (v1.2) | Both |  |
| Warrior Identity Scale [59] | Both | Skipped if participant was not/has not been in the U.S. military, or is/was in the Canadian military.  Items specific to Veterans were skipped if participant is on Active duty in the U.S. military. |
| Amputee Body Image Scale, Revised (ABIS-R) [60] | Both | Skipped if participant does not have history of limb loss/amputation.  Several items skipped if participant does not use a prosthetic device (abisr03, abisr05, abisr10, abisr12, abisr14).  One item skipped if participant is unable to walk (abisr13). |
| Trinity Amputation and Prosthesis Experience Scales – Revised (TAPES-R) Psychosocial Adjustment Subscale [61] | Both | Consisted of General Adjustment, Social Adjustment, and Adjustment to Limitations subscales (5 items each).  Skipped if participant does not use a prosthetic device. |

*Notes:* Block randomization was used to assign participants to one of two randomization groups (Arm 1 or 2).

***Supplemental Table 3. New LIMB-QOL Measure Score Means (SDs) by Injury Location***

|  | **Lower Extremity Injury** | | **Upper Extremity Injury** | | **Upper and Lower Injuries** | |
| --- | --- | --- | --- | --- | --- | --- |
| **Domain** | **n** | **M (SD)** | **n** | **M (SD)** | **n** | **M (SD)** |
| Satisfaction with Physical Fitness & Athleticism | 412 | 50.0 (9.9) | 113 | 51.3 (9.5) | 77 | 48.0 (8.7) |
| Satisfaction with Orthosis/Prosthesis | 345 | 51.2 (9.3) | 62 | 44.8 (9.8) | 64 | 48.3 (9.4) |
| Body Image | 412 | 50.5 (10.0) | 113 | 49.7 (8.7) | 77 | 47.5 (9.9) |
| Future Outlook | 412 | 49.8 (9.8) | 113 | 51.8 (9.9) | 77 | 48.1 (9.3) |
| Grief and Loss | 412 | 49.8 (9.6) | 113 | 49.8 (10.1) | 77 | 51.2 (9.9) |
| Health-Related Self-Efficacy | 408 | 50.1 (9.5) | 110 | 50.6 (9.5) | 74 | 48.8 (9.1) |
| Resilience | 412 | 49.8 (9.8) | 114 | 51.8 (10.2) | 77 | 48.5 (9.2) |
| Self-Esteem | 412 | 50.2 (10.0) | 113 | 51.0 (9.3) | 77 | 47.1 (9.5) |
| Weight Satisfaction | 412 | 50.3 (10.2) | 113 | 49.5 (8.6) | 77 | 49.0 (8.4) |
| Vocational Impact | 412 | 49.7 (9.3) | 113 | 49.5 (10.4) | 77 | 52.4 (9.3) |

***Supplemental Table 4. New LIMB-QOL Measure Score Means (SDs) by Limb Treatment Status***

|  | **Surgical**  **Preservation /**  **Reconstruction** | | **Limb Loss / Amputation** | |
| --- | --- | --- | --- | --- |
| **Domain** | **n** | **M (SD)** | **n** | **M (SD)** |
| Satisfaction with Physical Fitness & Athleticism | 184 | 48.0 (10.0) | 418 | 50.9 (9.5) |
| Satisfaction with Orthosis/Prosthesis | 101 | 45.9 (8.2) | 370 | 51.1 (9.7) |
| Body Image | 184 | 49.4 (9.5) | 418 | 50.2 (10.0) |
| Future Outlook | 184 | 49.9 (9.4) | 418 | 50.0 (10.0) |
| Grief and Loss | 184 | 51.5 (9.5) | 418 | 49.3 (9.8) |
| Health-Related Self-Efficacy | 180 | 49.5 (9.5) | 412 | 50.2 (9.4) |
| Resilience | 184 | 49.0 (9.4) | 419 | 50.4 (10.0) |
| Self-Esteem | 184 | 49.0 (9.1) | 418 | 50.4 (10.1) |
| Weight Satisfaction | 184 | 49.4 (9.5) | 418 | 50.2 (9.8) |
| Vocational Impact | 184 | 50.3 (9.4) | 418 | 49.9 (9.6) |
